# Mechanical properties of the everolimus-eluting bioresorbable vascular scaffold compared to the metallic everolimus-eluting stent

**DOI:** 10.1186/s12872-016-0296-1

**Published:** 2016-05-25

**Authors:** Daniel Dalos, Clemens Gangl, Christian Roth, Lisa Krenn, Sabine Scherzer, Markus Vertesich, Irene Lang, Gerald Maurer, Thomas Neunteufl, Rudolf Berger, Georg Delle-Karth

**Affiliations:** Department of Internal Medicine II, Division of Cardiology, Medical University of Vienna, Waehringer Guertel 18-20, 1090 Vienna, Austria; Department of Internal Medicine, Division of Cardiology, University Hospital Krems, Krems, Austria; Department of Internal Medicine, Division of Cardiology, Barmherzige Brüder Eisenstadt, Eisenstadt, Austria; Department of Internal Medicine, Division of Cardiology, Krankenhaus Hietzing, Vienna, Austria

**Keywords:** Bioresorbable Scaffold, Drug-Eluting Stent, Optical Coherence Tomography

## Abstract

**Background:**

Everolimus-eluting bioresorbable vascular scaffolds (BVS) represent an innovative treatment option for coronary artery disease. Clinical and angiographic results seem promising, however, data on its immediate procedural performance are still scarce. The aim of our study was to assess the mechanical properties of BVS by Optical Coherence Tomography (OCT) in clinical routine.

**Methods:**

Post-implantation OCT images of 40 BVS were retrospectively compared to those of 40 metallic everolimus-eluting stents (EES). Post-procedural device related morphological features were assessed. This included incidences of gross underexpansion and the stent eccentricity index (SEI, minimum/maximum diameter) as a measure for focal radial strength.

**Results:**

Patients receiving BVS were younger than those with EES (54.0 ± 11.2 years versus 61.7 ± 11.4 years, *p* = 0.012), the remaining baseline, vessel and lesion characteristics were comparable between groups. Lesion pre-dilatation was more frequently performed and inflation time was longer in the BVS than in the EES group (*n* = 34 versus *n* = 23, *p* = 0.006 and 44.2 ± 12.8 versus 25.6 ± 8.4 seconds, *p* < 0.001, respectively). There were no significant differences in maximal inflation pressures and post-dilatation frequencies with non-compliant balloons between groups. Whereas gross device underexpansion was not significantly different, SEI was significantly lower in the BVS group (*n* = 12 (30 %) versus *n* = 14 (35 %), *p* = 0.812 and 0.69 ± 0.08 versus 0.76 ± 0.09, *p* < 0.001, respectively). There was no difference in major adverse cardiac event-rate at six months.

**Conclusion:**

Our data show that focal radial expansion was significantly reduced in BVS compared to EES in a clinical routine setting using no routine post-dilatation protocol. Whether these findings have impact on scaffold mid-term results as well as on clinical outcome has to be investigated in larger, randomized trials.

## Background

Drug eluting stents (DES) have shown to be highly effective in the treatment of patients with coronary artery disease [1–3] as neointimal hyperplasia after a vascular injury was reduced compared to when bare metal stents were used [2, 3]. Nevertheless, delayed or absent strut endothelialization, persistent or acquired malapposition and neoatherosclerosis of DES contribute to late stent failure rates which are in the range of 1-2 % a year within the first three years after implantation [4–6]. In addition stent fractures especially at hinge points of the coronary vessels and the lack of adaptive remodelling processes in the artery wall can contribute to late events.

Bioresorbable vascular scaffolds (BVS) were developed in order to reduce those potential adverse events after a coronary intervention. After the bioresorption process is completed there will not be any potential triggers for late adverse events [7]. In contrast to vessels caged by metallic stents, vessels transiently scaffolded by bioresorbable materials are able to perform vasoconstriction and vasodilation and therefore could also contribute to better symptom control in patients with coronary artery disease [8–11]. It was also shown that BVS are characterized by a better conformability to the vessel compared to metallic stents [12]. On the other hand it is still unclear if the radial strength provided by BVS is sufficient throughout various clinical scenarios. It has been shown that metallic stents generate a larger acute lumen gain compared to BVS, but scaffold/stent type was not predictive for acute recoil [13]. Intravascular imaging data describing device strength and expansion are still scarce. The aim of the present study was to assess the mechanical properties of BVS by Optical Coherence Tomography (OCT) in clinical routine.

## Methods

### Patients

Between March and June 2013, 26 consecutive patients underwent OCT immediately after implantation of 40 BVS (Absorb, Abbott Vascular, Santa Clara, CA, USA). Elective patients as well as patients presenting with acute coronary syndrome (ACS) were included. The OCT data of these patients were retrospectively compared with those of 34 consecutive patients after implantation of 40 metallic everolimus-eluting stents (EES, Xience, Abbott Vascular^,^ Santa Clara, CA, USA).

Patient characteristics were collected from the medical records of each patient, device characteristics and deployment strategies were collected from the database of the cardiac catheter laboratory. This study was approved by the ethics committee of the Medical University of Vienna and all patients gave their written informed consent.

### Definitions

*Stent eccentricity index (SEI)*: at the site of minimal lumen area (MLA) stent eccentricity index, defined as the ratio between minimal and maximal diameter, was calculated [14].

*Stent symmetry index (SSI)*: at the site of MLA stent symmetry index, defined as (maximal-minimal diameter)/maximal diameter, was calculated [14].

*Underexpansion*: a stent was considered underexpanded if MLA was ≤ 80 % of average reference lumen area [15].

*Plaque characteristics*: plaque type was determined at proximal and distal stent ending, as well as at MLA and was considered to be either lipid-rich, fibrous or fibro-calcific [16].

*Incomplete stent apposition (ISA)*: struts were considered incompletely apposed when they were separated from the underlying vessel wall in case of BVS [17] or when the axial distance between strut's surface and the luminal surface was greater than the strut thickness in case of EES [18].

### OCT - acquisition and analysis

Patients were pre-treated with a dual antiplatelet therapy, a weight-adjusted intravenous bolus of unfractionated heparin, and 200 μg intracoronary nitroglycerine. The OCT images were obtained using a frequency domain (FD) - OCT system (LightLab Imaging, Inc., Westford, MS, USA). The FD-OCT imaging catheters were delivered over a 0.014-inch (0.0356 cm) guide wire through a 6-F guiding catheter. Images were acquired using a motorized pullback system at a speed of 36 mm/s during a flush of 4 to 6 ml/s of iso-osmolar contrast (Iodixanol 320, VisipaqueTM, GE Health Care, Cork, Ireland) through the guiding catheter to replace the blood flow and permit the visualization of the stented segment and intima-lumen interface. Whenever the stented segment was too long to be completely imaged in a single pullback, the image acquisition was repeated from the same position during a second contrast injection. Anatomic landmarks such as side branches, calcifications or stent overlap segments were used for longitudinal view orientation.

OCT imaging was performed after what was deemed to be an angiographically successful intervention at the operator’s discretion. All OCT frames were digitally stored and independently analyzed using an offline software (LightLab Console) by one operator, who is experienced in and familiar with assessing OCT images. Cross-sections within the stented segment were analyzed every frame.

### Statistical analysis

The statistical analysis was conducted with SPSS® Statistics 21.0 (SPSS Inc, Chigago, USA). Quantitative data were presented as mean ± standard deviation. Qualitative data were presented as frequencies. Categorical variables were assessed by *χ*^2^ statistics and Fisher's exact test. Continuous variables were compared using an unpaired *t*-test. A *p*-value <0.05 was considered statistically significant.

## Results

Patients receiving BVS were younger than those with EES (54.0 ± 11.2 years vs. 61.7 ± 11.4 years, *p* = 0.012) and were smokers less frequently. The remaining baseline characteristics were comparable between the two groups (Table 1). The majority of treated lesions in both groups were simple lesions (type B1, 62 % in BVS vs. 70 % in EES group, *p* = 0.407).

**Table 1:** Baseline characteristics

	*BVS (n = 26)*	*EES (n = 34)*	*P-value*
Age, years	54.0 ± 11.2	61.7 ± 11.4	0.012
Male, n (%)	19 (73)	30 (88)	0.122
*Diabetes*			
No, n (%)	18 (69)	25 (74)	
NIDDM, n (%)	5 (19)	9 (26)	0.117
IDDM, n (%)	3 (12)	0	
Hypertension, n (%)	15 (58)	25 (74)	0.156
Hyperlipidemia, n (%)	17 (65)	21 (62)	0.494
Positive family history, n (%)	6 (23)	10 (29)	0.402
*Smoking*			
No, n (%)	15 (58)	9 (26)	
Ex, n (%)	2 (8)	13 (38)	0.011
Current, n (%)	9 (34)	12 (36)	
*Indication for PCI*			
ACS - STEMI, n (%)	3 (12)	1 (3)	
ACS - NSTEMI, n (%)	7 (27)	6 (18)	0.237
Non-ACS elective, n (%)	16 (61)	27 (79)	
LAD, n (%)	19 (73)	19 (56)	
CX, n (%)	3 (11)	9 (26)	0.261
RCA, n (%)	4 (15)	5 (15)	
Ramus intermedius, n (%)	1 (5)	0	
*Lesion classification*			
Type B1, n (%)	16 (62)	24 (70)	
Type B2, n (%)	5 (19)	5 (15)	0.407
Type C, n (%)	5 (19)	5 (15)	

*ACS* Acute Coronary Syndrome, *IDDM* Insulin Dependent Diabetes Mellitus, *NIDDM* Non-Insulin Dependent Diabetes Mellitus, *NSTEMI* Non-ST Elevation Myocardial Infarction, *STEMI* ST Elevation Myocardial Infarction

Device and procedural characteristics are depicted in Table 2. Pre-dilatation was more frequently performed before BVS implantation and the diameter of the pre-dilatation balloon was larger in the BVS group (85 % vs. 58 %, *p* = 0.006 and 2.64 ± 0.51 mm vs. 2.34 ± 0.46 mm, *p* = 0.026, respectively), while BVS diameters were larger compared to those of EES (3.23 ± 0.34 mm vs. 3.07 ± 0.54 mm, *p* = 0.124). Lesion diameter stenosis was less severe in the BVS group and the inflation time was longer (81 ± 14 % vs. 89 ± 14 %, *p* = 0.023 and 44 ± 13 sec vs. 26 ± 8 sec, *p* < 0.001, respectively).

**Table 2:** Device and procedural characteristics

	*BVS (n = 40)*	*EES (n = 40)*	*P-value*
Nominal diameter, mm	3.23 ± 0.34	3.07 ± 0.54	0.124
Length, mm	21.80 ± 5.62	21.13 ± 9.21	0.693
Stenosis, %	81 ± 14	89 ± 14	0.023
Inflation time, sec	44 ± 13	26 ± 8	<0.001
Inflation pressure, atm	13 ± 3	13 ± 3	0.799
Predilatation, n (%)	34 (85)	23 (58)	0.006
Predilatation-ballon diameter, mm	2.64 ± 0.51	2.34 ± 0.46	0.026
Predilatation-ballon inflation pressure, atm	13 ± 2	12 ± 3	0.266
Postdilatation, n (%)	22 (55)	27 (68)	0.179
Postdilatation-ballon diameter, mm	3.41 ± 0.47	3.27 ± 0.60	0.380
Postdilatation-ballon inflation pressure, atm	15 ± 3	16 ± 4	0.282
Postdilatation with NC-ballon, n (%)	21 (53)	16 (40)	0.321
Contrast volume, ml	215.2 ± 150.9	213.9 ± 96.8	0.963
Radiation time, min	12.1 ± 9.2	11.8 ± 6.5	0.874

*CX* Circumflex Artery, *LAD* Left Anterior Descending Artery, *LM* Left Main, *NC* Non-Compliant, *RCA* Right Coronary Artery

### OCT results

OCT analysis (Table 3) showed comparable plaque compositions between the two groups at the proximal and distal stent endings as well as at the point of minimal lumen area. Although there were no statistically significant differences in the minimal lumen area and in the incidence of device underexpansion between the groups (6.38 ± 2.00 mm^2^ vs. 6.42 ± 2.42 mm^2^, *p* = 0.662 and 30 % vs. 35 %, *p* = 0.812, respectively) the SEI at the site of the minimal lumen area was significantly lower in the BVS group compared to EES (0.69 ± 0.08 vs. 0.76 ± 0.09, *p* < 0.001). SSI was significantly higher in BVS compared to EES (0.30 ± 0.09 vs. 0.23 ± 0.09, *p* < 0.001).

**Table 3:** OCT analysis

	*BVS (n = 40)*	*EES (n = 40)*	*P-value*
*Plaque type distal stent ending*			
Lipid rich, n (%)	21 (53)	20 (50)	
Fibrous, n (%)	17 (42)	15 (38)	0.493
Fibro-calcific, n (%)	2 (5)	5 (12)	
*Plaque type proximal stent ending*			
Lipid rich, n (%)	23 (58)	15 (38)	
Fibrous, n (%)	8 (20)	12 (30)	0.114
Fibro-calcific, n (%)	9 (22)	13 (32)	
*Plaque type MLA*			
Lipid rich, n (%)	20 (50)	18 (45)	
Fibrous, n (%)	8 (20)	8 (20)	0.826
Fibro-calcific, n (%)	8 (20)	12 (30)	
Plaque rupture - hematoma, n (%)	4 (10)	2 (5)	
Reference vessel area distal, mm^2^	6.40 ± 2.11	6.49 ± 2.57	0.879
Reference vessel diameter distal, mm	2.78 ± 0.49	2.80 ± 0.64	0.854
Reference vessel area proximal, mm^2^	8.98 ± 2.55	9.43 ± 4.15	0.560
Reference vessel diameter proximal, mm	3.37 ± 0.51	3.44 ± 0.76	0.631
Reference vessel area, mm^2^	7.69 ± 2.17	7.96 ± 3.30	0.668
Reference vessel diameter, mm	3.07 ± 0.44	3.09 ± 0.62	0.834
Distal stent ending area, mm^2^	6.77 ± 2.34	6.97 ± 2.75	0.721
Distal stent ending diameter, mm	2.88 ± 0.53	2.92 ± 0.56	0.750
Proximal stent ending area, mm^2^	7.48 ± 2.35	8.62 ± 3.51	0.092
Proximal stent ending diameter, mm	3.04 ± 0.49	3.25 ± 0.63	0.102
Difference nominal diameter-proximal stent ending diameter, mm	0.19 ± 0.37	- 0.18 ± 0.52	<0.001
Mean stent area, mm^2^	7.12 ± 2.20	7.79 ± 2.30	0.257
Mean stent diameter, mm	2.96 ± 0.48	3.08 ± 0.57	0.984
Minimal lumen area, mm^2^	6.38 ± 2.00	6.42 ± 2.42	0.662
Minimal stent diameter, mm	2.46 ± 0.43	2.53 ± 0.54	0.537
Maximal stent diameter, mm	3.53 ± 0.52	3.31 ± 0.64	0.085
Stent eccentricity index	0.69 ± 0.08	0.76 ± 0.09	<0.001
Stent symmetry index	0.30 ± 0.09	0.23 ± 0.09	<0.001
RAS, %	16.99 ± 12.86	17.70 ± 11.50	0.795
Rate of ISA, %	3.4 ± 1.9	2.9 ± 1.9	0.720
Underexpansion, n (%)	12 (30)	14 (35)	0.812
Underexpansion & hematoma, n (%)	1 (3)	1 (3)	1.000
Stent fracture, n (%)	3 (8)	0	0.241

*ISA* Incomplete Stent Apposition, *MLA* Minimal Lumen Area, *RAS* Residual Area Stenosis

### Clinical follow-up

After six months there was no detectable difference in major adverse cardiac event (MACE) rate between groups (Table 4). One patient in the BVS group underwent angiography after three months because of recurrent angina. Subacute thrombotic lesions were angiographically revealed and thrombus aspiration following dilatation with a non-compliant balloon was performed. One patient in the EES group was re-admitted due to unstable angina after 45 days. Angiography showed an intima-flap at the proximal stent ending which resulted in another overlapping stent-implantation. Another EES patient underwent elective angiography after three months. Although he presented free of any symptoms EES showed significant in-stent-restenosis which was fixed by drug-eluting balloon dilatation.

**Table 4:** Six months clinical follow-up

	*BVS (n = 26)*	*EES (n = 34)*	*P-value*
MACE, n (%)	1 (4)	2 (6)	1.000
Death, n (%)	0	0	
MI, n (%)	0	0	
TV-Revasc., n (%)	1 (4)	2 (6)	1.000
Non-TV-Revasc. n (%)	0	0	

*MACE* Major Adverse Cardiac Event, *MI* Myocardial Infarction, *TV* Target Vessel, Revasc - Revascularization

## Discussion

Whereas BVS were rapidly adopted in clinical routine, prospective data especially with regard to complex lesions are still rare [19–21]. Recent data suggest that acute and late stent thrombosis are more frequent in BVS compared to DES [22], which is potentially associated with the unique mechanical properties of BVS. It has been shown that OCT is a valuable tool in determining post-procedural success with both BVS and DES as it potentially reduces late complications caused by mechanical shortcomings of the devices not detected when using angiography. This is the first OCT study comparing BVS and EES in a clinical routine setting.

**Fig. 1:**
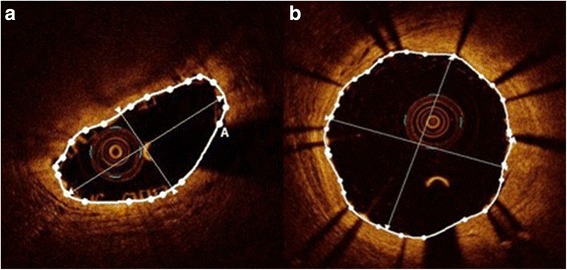
Automatic measurements performed at site of minimal lumen area in BVS (**a**) and EES (**b**)

The main finding of this study was that in a routine setting using no post-dilatation protocol, although gross device expansion of BVS and EES was comparable, device expansion uniformity of BVS, defined by SEI at the point of MLA, was significantly lower, whereas SSI was higher in BVS compared to EES (Fig. 1).

**Fig. 2:**
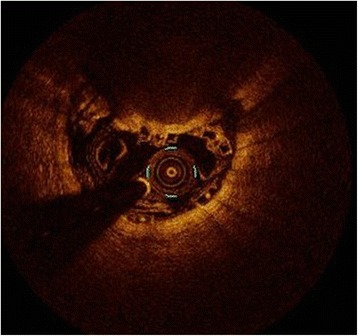
Reduced radial expansion of BVS in lesions with intramural hemorrhage

This lower device expansion can potentially contribute to an alteration in blood flow dynamics and can therefore represent a source for acute and subacute post-procedural complications as shown by Otake and coworkers [23]. Interestingly, this finding contradicts the study of Mattesini [14] but is in line with that of Brugaletta and coworkers in a substudy of the Absorb cohort B trial [24]. The most plausible explanation is that in the study of Mattesini a more aggressive vessel preparation with a balloon/artery ratio 1:1 and a stringent OCT guided post-dilatation balloon inflation strategy with non-compliant balloons were performed. In our study pre-dilatation was performed with in average 0.5 mm undersized balloons in 85 % of all cases and post-dilatation with NC-balloons was done only in 55 % of BVS implantations which is closer to the treatment procedure used in in the Absorb cohort B trial. Accordingly post-dilatation was performed in only 50 % of cases in the Ghost-EU trial, which included 1189 patients [25]. It is noteworthy that especially in the early phase the incidences of scaffold thrombosis were more frequent than anticipated in this study, which may be influenced by the low rates of post-dilatation (<40 %) in these patients with BVS failure. Although the lack of a stringent post-dilatation protocol is the most plausible cause for the discussed differences in SEI other contributing factors have to be considered. In contrast to our study, in which patients with ACS represented > 1/3 of the study population, they were not included in the study of Mattesini. In patients presenting with ACS, previous imaging analysis found more complex lesion morphologies when compared to patients with stable CAD [26]: Especially developing intramural hemorrhages is more common in acute lesions and because of the limited radial strength of BVS [13] they might not be able to withstand the consecutively increased vessel wall resistance (Fig. 2). The different post-procedural stent geometry was also supported by significant differences in the nominal vessel/proximal device ending diameter, where the proximal BVS ending was in average smaller and the proximal EES was in average larger than the native vessel diameter. This finding is in accordance with the study of Mattesini, where ISA at the proximal edge was more frequently observed in the BVS group. Although the clinical significance of ISA is not definitely determined, it has been associated with stent thrombosis in IVUS studies [27, 28]. In addition protruding struts at the proximal edge may complicate the advancement of other devices distal of the stent. However compared to DES the bioabsorbable technology has the potential advantage of ISA being at least only a temporary problem.

### Limitations

This is a retrospective, single center, non-randomized observational study in a limited number of patients. Although patient characteristics were well matched no propensity adjustments have been performed. Final OCT assessment was performed, when the operators deemed to be angiographically successful, which provides a potential bias.

Nevertheless in the learning curve of a new technology it is of high value to identify potential safety concerns additionally to large randomized trials in clinical studies. In our routine setting we could reveal significant differences in the post-procedural geometry between BVS and EES using OCT. Whether these differences may contribute to the somewhat higher stent thrombosis rates observed in other series remains unclear. To avoid inappropriate BVS expansion it seems advisable to incorporate routine post-dilatation with NC-balloons in the procedural protocol. The role of aggressive pre-dilatation with its potential complications remains a matter of debate and the use of intravascular imaging may further delineate the appropriate use of BVS.

## Conclusion

Although minimal lumen areas and rates of device underexpansion were comparable between BVS and EES, local radial expansion is significantly reduced in BVS in a clinical routine setting using no post-dilatation protocol. The clinical importance of this finding remains unclear and has to be evaluated in larger, randomized trials. However, a lower uniform expansion of the BVS could contribute to clinical events such as scaffold thrombosis and should be considered when implanting these devices. Considering the relatively low number of post-dilatations with non-compliant balloons in the BVS group our data further suggest that routine post-dilatation should be strongly considered.

## Abbreviations

ACS, acute coronary syndrome; BVS, bioresorbable vascular scaffold; CAD, coronary artery disease; DES, drug eluting stent; EES; everolimus eluting stent; FD, frequency domain; ISA, incomplete stent apposition; IVUS, intra vascular ultra sound, MACE, major adverse cardiovascular event; MLA, minimal lumen area; NC, non compliant; OCT, optical coherence tomography; SEI, stent eccentricity index; SSI, stent symmetry index.
